# BenchAMRking: a Galaxy-based platform for illustrating the major issues associated with current antimicrobial resistance (AMR) gene prediction workflows

**DOI:** 10.1186/s12864-024-11158-5

**Published:** 2025-01-10

**Authors:** Nikolaos Strepis, Dennis Dollee, Donny Vrins, Kevin Vanneste, Bert Bogaerts, Catherine Carrillo, Amrita Bharat, Kristy Horan, Norelle L. Sherry, Torsten Seemann, Benjamin P. Howden, Saskia Hiltemann, Leonid Chindelevitch, Andrew P. Stubbs, John P. Hays

**Affiliations:** 1 Department of Medical Microbiology and Infectious Diseases, Erasmus University Medical Centre (Erasmus MC), Rotterdam, The Netherlands; 2 Department of Pathology and Clinical Bioinformatics, Erasmus University Medical Centre (Erasmus MC), Rotterdam, The Netherlands; 3 Transversal activities in Applied Genomics, Sciensano, Brussels, Belgium; 4 Canadian Food Inspection Agency, Ottawa, ON K1A 0Y9 Canada; 5 National Microbiology Laboratory, Public Health Agency of Canada, Winnipeg, MB R3E 3R2 Canada; 6 Microbiological Diagnostic Unit Public Health Laboratory (MDU-PHL), Department of Microbiology & Immunology, University of Melbourne at the Peter Doherty Institute for Infection & Immunity, Melbourne, VIC Australia; 7 Institute of Pharmaceutical Sciences, Faculty of Chemistry and Pharmacy, University of Freiburg, 79104 Freiburg, Germany; 8 MRC Centre for Global Infectious Disease Analysis, Imperial College London, London, England, UK

**Keywords:** Antimicrobial resistance, Microbial whole genome sequencing, Benchmarking, Galaxy, Workflows

## Abstract

**Background:**

The Joint Programming Initiative on Antimicrobial Resistance (JPIAMR) networks ‘Seq4AMR’ and ‘B2B2B AMR Dx’ were established to promote collaboration between microbial whole genome sequencing (WGS) and antimicrobial resistance (AMR) stakeholders. A key topic discussed was the frequent variability in results obtained between different microbial WGS-related AMR gene prediction workflows. Further, comparative benchmarking studies are difficult to perform due to differences in AMR gene prediction accuracy and a lack of agreement in the naming of AMR genes (semantic conformity) for the results obtained. To illustrate this problem, and as a capacity-building exercise to encourage stakeholder involvement, a comparative Galaxy-based BenchAMRking platform was developed and validated using datasets from bacterial species with PCR-verified AMR gene presence or absence information from abritAMR.

**Results:**

The Galaxy-based BenchAMRking platform (https://erasmusmc-bioinformatics.github.io/benchAMRking/) specifically focusses on the steps involved in identifying AMR genes from raw reads and sequence assemblies. The platform currently comprises four well-characterised and published workflows that have previously been used to identify AMR genes using WGS data from several different bacterial species. These four workflows, which include the ISO certified abritAMR workflow, make use of different computational tools (or tool versions), and interrogate different AMR gene sequence databases. By utilising their own data, users can investigate potential AMR gene-calling problems associated with their own in silico workflows/protocols, with a potential use case outlined in this publication.

**Conclusions:**

BenchAMRking is a Galaxy-based comparison platform where users can access, visualise, and explore some of the major discrepancies associated with AMR gene prediction from microbial WGS data.

**Supplementary Information:**

The online version contains supplementary material available at 10.1186/s12864-024-11158-5.

## Background

Antimicrobial resistance (AMR) represents a current global pandemic that detrimentally affects hospitalized patients, community-based care, healthcare system economics and a variety of One Health ecosystems, including foodstuffs, (domestic) animal health and the environment [1]. Furthermore, AMR is facilitated by a variety of factors, including a lack of implementation of infection prevention protocols, inappropriate antibiotic use, the slow development of new (alternative) antimicrobials and the time-consuming detection of AMR phenotypes.

Until recently, the detection of AMR in microbial isolates was almost solely based on phenotypic testing, which tends to provide accurate mechanism-independent results that can be confidently used by clinicians in their antimicrobial prescribing decisions. However, techniques involving mass spectrometry and genotype-to-phenotype AMR gene prediction are gaining in importance [2]. For example, whole genome sequencing of bacteria is frequently incorporated into infectious epidemiology studies and infection prevention programs, as well as in the genotype-to-phenotype prediction of AMR. Although concordance between existing genotype-to-phenotype AMR prediction workflows is generally good, a successful implementation in the clinical setting requires global agreement on standardisation, quality control parameters and validation for genotype-to-phenotype prediction, which begins with the accurate identification of AMR genes from WGS data [3, 4]. This process includes two major steps: (1) the generation of lists of AMR genes from available sequence data and (2) the prediction of AMR phenotypes based on the lists of AMR genes. In this respect, the current number and variety of AMR gene prediction workflows, tools and tool versions is limiting the re-use of both the data and workflows that have previously been published. Therefore, the authors’ aim is to provide AMR researchers with easy access to standardised and validated AMR gene prediction workflows, which they could use with confidence when predicting AMR genes in their own One Health ecosystems. The result is BenchAMRking, a reusable Galaxy-based platform for AMR detection workflows that can deliver curated data and ground truth results for use by end users that are not familiar with deploying or using command line applications. The BenchAMRking platform includes a set of Galaxy workflows based on previously published AMR analysis workflows using the associated data and ground truth results to validate these workflows within a single resource. Currently, BenchAMRking allows both multi-species AMR gene prediction based on abritAMR [5], and species-specific AMR gene prediction originally used for *Escherichia coli* [6] and *Salmonella spp.* found in food [7] and human patients [8], respectively. The workflows represent the ground truth in a comprehensive output format, and their Galaxy versions are available from the Erasmus MC GitHub and Workflow hub. The use of Galaxy and Workflow hub ensures the sustainability, reproducibility and reusability of these tools and associated data, thereby helping mitigate against application obsolescence.

The analytical and interpretation-based problems associated with predicting AMR phenotypes from AMR gene-based data are not addressed by BenchAMRking, as this subject requires an additional level of complexity. Further, if the correct identification of AMR genes is challenging, then those challenges will also be likely to affect the downstream prediction of AMR phenotypes.

## Implementation

### Tools

**Fig. 1:**
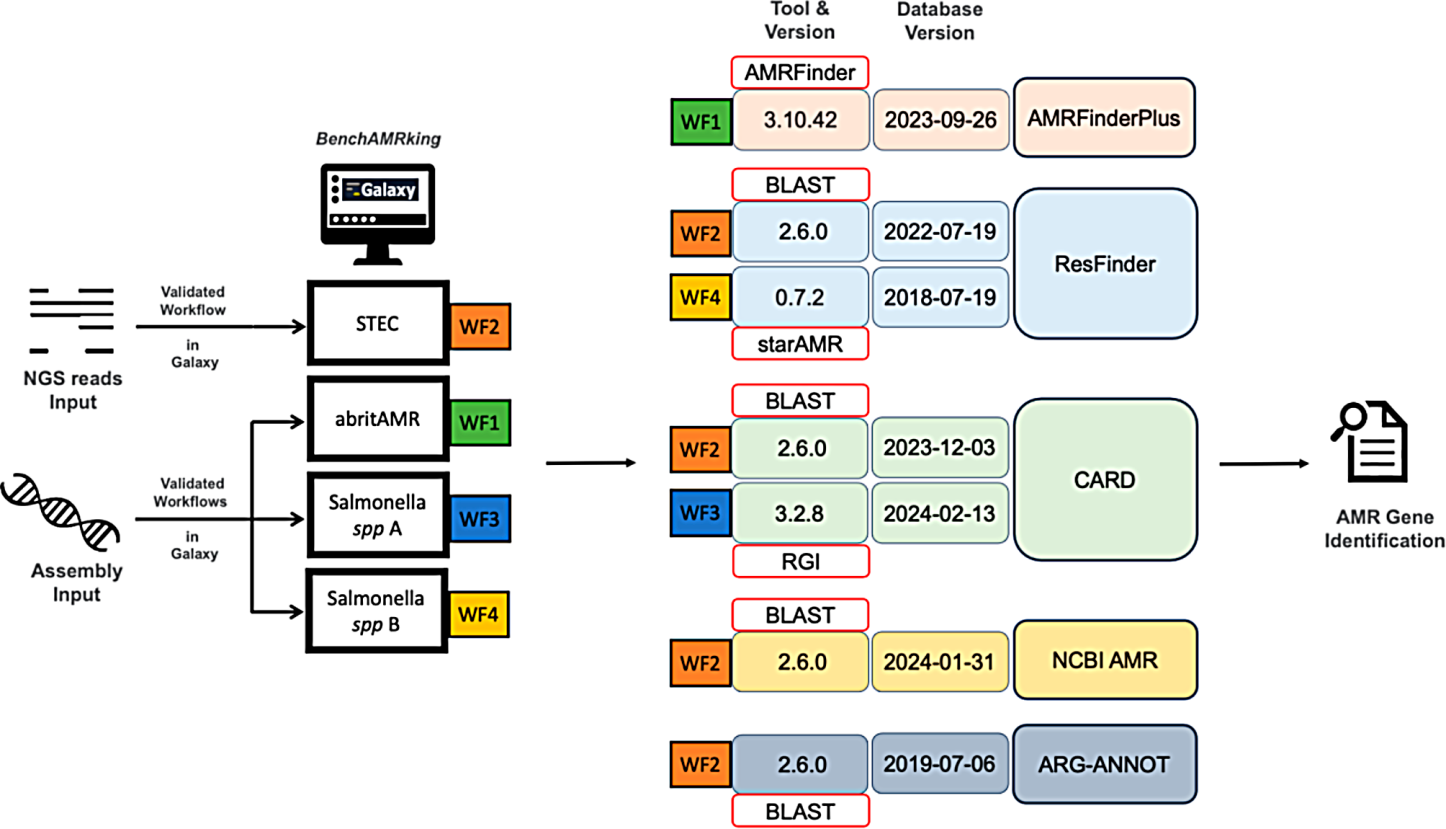
Overview of BenchAMRking platform and workflows. Selected AMR gene prediction workflows (WFs) are translated into Galaxy workflows and stored in Workflow Hub. Researchers can load them into a Galaxy instance of their choice and either use the published data to reproduce the results or analyse their own data. Published *Salmonella* spp A WF3 (from broiler chickens) and published *Salmonella* spp B WF4 (from human infections) represent different workflows

We have integrated a diverse collection of four previously published AMR gene prediction workflows into Galaxy for comparative benchmarking via the BenchAMRking platform (Fig. 1; Tables 1 and 2). The platform can be found at https://erasmusmc-bioinformatics.github.io/benchAMRking/, including brief instructions on its use. The output of the BenchAMRking platform may be visualised using the R-based Confusion Matrix and Heatmap scripts available from the BenchAMRking website. The workflows included in the BenchAMRking platform enable non-bioinformatics-trained researchers to perform extensive genomics analysis using short read sequence data, without the need for any coding. All workflows and their dependencies are installed on Galaxy and are managed by the Bioconda framework for dependency management. BenchAMRking workflows and their dependencies are available from the Bioconda Conda channel. The Galaxy wrappers were developed in GitHub for testing and have been made available on the Galaxy ToolShed.

**Table 1:** Version and licence information for the different workflow tools used in the BenchAMRking platform

Tool	Version	Licence
abritAMR	1.0.14	Creative Commons Attribution 4.0 International
RGI	5.2.1	custom license - free academic, government, non-profit use
SeqSero2	1.2.1	GNU GPL v2.0
BBTools	39.01	MIT License
SRST2	0.2.0	BSD License
hamronize	1.0.3	GNU LGPL v3.0
SPAdes	v3.15.5	GNU GPL v2.0
SKESA	3.0.0	Public Domain
pilon	1.1.0	GNU GPL v2.0
sistr	1.1.1	Apache-2.0 license
MOB-Recon	3.0.3	Apache-2.0 license
Shovill	1.0.4	GPL-3.0 license
staramr	0.8.0	Apache-2.0 license

**Table 2:** Database version information for the different workflow tools used in the BenchAMRking platform

Workflow	Database	Database version
WF1	AMRFinderPlus	2023-09-26
WF2	ResFinder	2022-07-19
WF2	CARD	2023-12-03
WF2	NCBI AMR	2024-01-31
WF2	ARG-ANNOT	2019-07-06
WF3	CARD	2024-02-13
WF4	ResFinder	2018-07-19

### Workflows

We have integrated four published WGS-AMR genotype prediction workflows (WF1-WF4) into Galaxy (Table 3). These workflows utilise a variety of bioinformatics applications copied from the original publications in which they were defined i.e., references WF1 [5]; WF2 [6]; WF3 [7] and WF4 [8]. In this publication, the ground truth for accurate AMR gene prediction is taken as the results obtained from the ISO certified abritAMR workflow (WF1). The replicated workflow data and results are all accessible at the BenchAMRking website (https://erasmusmc-bioinformatics.github.io/benchAMRking/ viaWorkflowHub (https://workflowhub.eu/). We note that a systematic review of available workflows was not performed when choosing the workflows used in BenchAMRking. Instead, a simple search of existing literature was made for workflows that met the criteria mentioned above and described below (WF1 and WF4). WF2 and WF3 are workflows that also meet these criteria and are currently in use by one or more partners of the ‘Seq4AMR’ and ‘B2B2B AMR Dx’ networks. Additionally, publications validated their workflows with clinical or surveillance isolates and the workflows contain most known AMR genes.

**Table 3:** Workflow availability

Workflow	WorkflowHub ID
**WF1**: abritAMR Multi-species [5]	https://workflowhub.eu/workflows/634
**WF2**: Sciensano *Escherichia coli* [6]	https://workflowhub.eu/workflows/644
**WF3**: CFIA Food *Salmonella* spp. [7]	https://workflowhub.eu/workflows/407
**WF4** : Staramr Human Health *Salmonella* spp. [8]	https://workflowhub.eu/workflows/470

WF: workflow

The current workflows are supported by publications that include validated datasets. The tool and versions used in this publication are shown in Fig. 1 and provided in a machine-readable format in Tables 1 and 2, respectively. Users should be aware that the results of the workflows might change when newer versions of tools or databases are implemented in future version of the original workflows (we used the versions listed in the relevant publications). Descriptions of the individual workflows are given below.

#### WF1: ISO abritAMR

An AMR detection and reporting workflow (certified to ISO standards in the originating laboratory), based on the AMRFinderPlus tool and further optimized for clinical use. AMR prediction is based on the AMRFinderPlus database, and reports customized for clinical and public health microbiology applications are generated with an enhanced database to classify AMR mechanisms, and reports filtered to contain the most relevant AMR mechanisms. An additional module provides inferred phenotype reports for *Salmonella* spp. An extensive validation dataset is provided including PCR data and synthetic genomic data across 42 species. The workflow was validated with 1,184 bacterial isolates (42 species) [5].

#### WF2: Sciensano

This workflow uses multiple tools that perform read trimming, genome assembly, contamination checks, quality control of reads, plasmid detection, sequence typing, serotype determination, virulence factors identification and AMR characterization (against the NCBI NDARO, ResFinder and PointFinder databases). The database used for AMR gene prediction are ResFinder, CARD, ARG-annot and NDARO. The data for this workflow included 137 Shiga toxin-producing *E. coli i*solates from human faeces and various food matrices that were tested with disc diffusion or PCR-based methods [6].

#### WF3: CFIA

This workflow is based on multiple tools that include quality control and read trimming, genome assembly, plasmid prediction and serotype prediction for *Salmonella* spp. genomes. The AMR prediction database is based on the CARD database. All results are subsequently standardised using the hAMRonization tool. This workflow was validated using phenotypic verification of AMR in isolates, which were performed using broth micro dilutions [7].

#### WF4: Staramr

This workflow is based on staramr, a tool for genotypic AMR prediction based on the Centre for Genomic Epidemiology’s ResFinder, PointFinder, and PlasmidFinder databases as well as PubMLST databases. Validation of the workflow was based on AMR phenotypic broth micro dilution of 1,321 *Salmonella enterica* isolates from the Canadian Integrated Program for Antimicrobial Resistance Surveillance (CIPARS) [8].

### Experiments

**Fig. 2:**
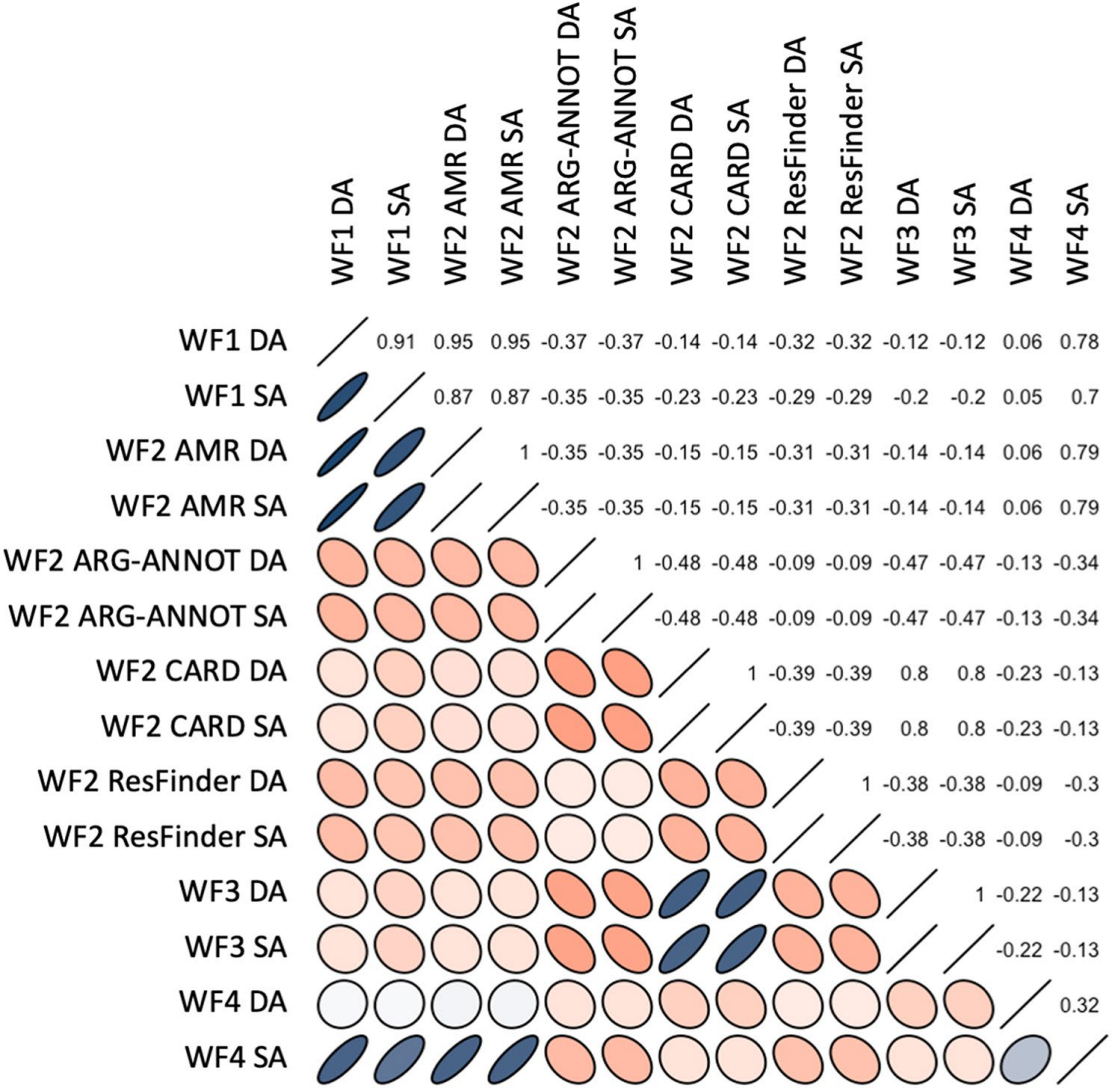
Correlation matrix of AMR gene presence/absence vectors among different workflows included in BenchAMRking. WF1 - AbritAMR; WF2 - Sciensano; WF3 - CFIA; WF4 - Staramr. Numbers on the top right indicate the correlation among workflows. Colour indicates a positive (red) or negative (blue) correlation, and shape indicates the strength of correlation. The more circular the shape, the stronger the correlation; the more oval the shape, the weaker the correlation. SA: same assembler; DA: different assembler (part of AMR identification and input of BenchAMRking). The supplemental data for the heatmaps are both the binary and identity excel files in the scripts repository at https://github.com/ErasmusMC-Bioinformatics/BenchAMRking-scripts/tree/main

**Fig. 3:**
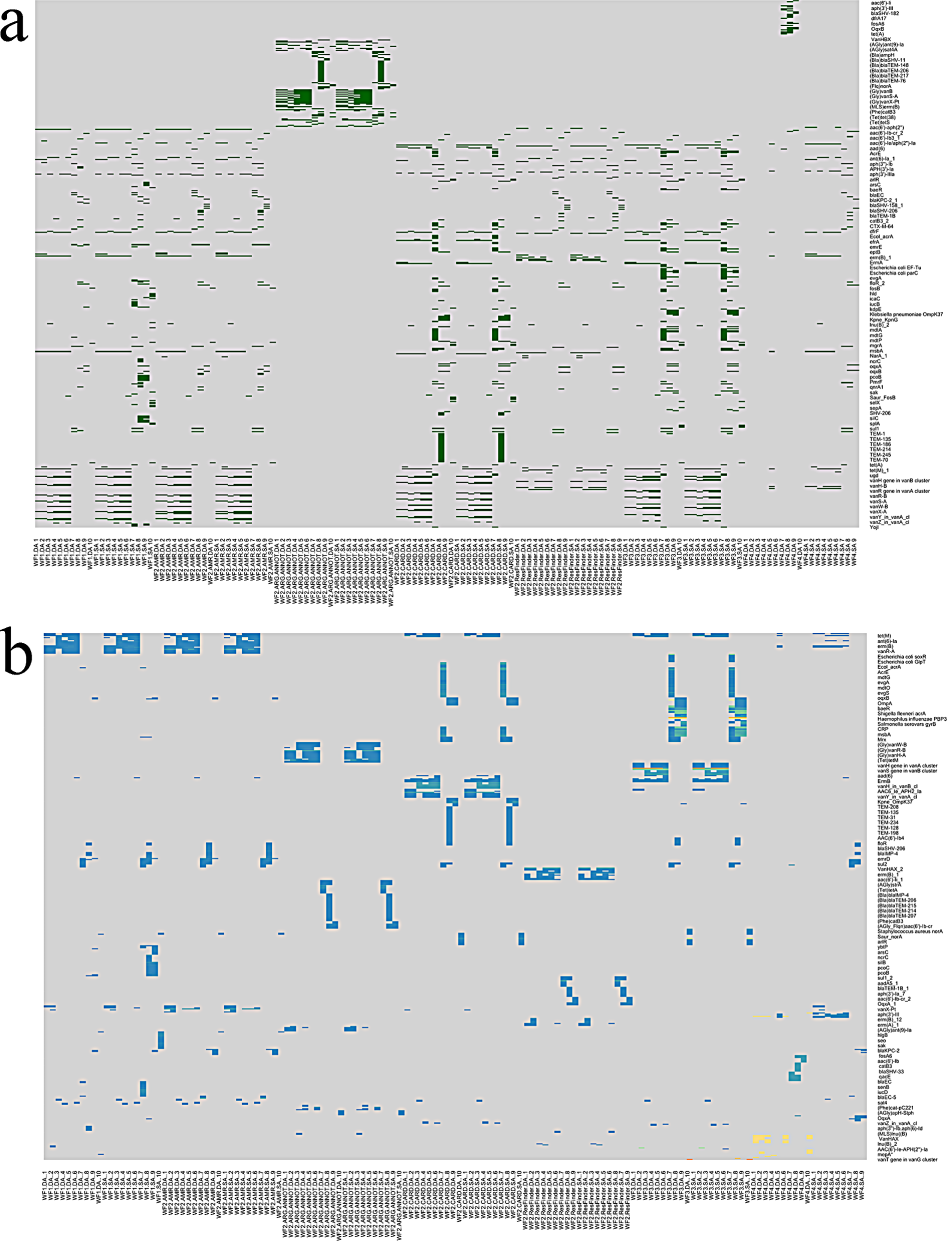
(**a**) Heatmap representation of the relationships of AMR genes detected in the workflows included in BenchAMRking. Green colour represents gene presence/absence. AMR genes are clustered based on identification by different workflows. SA: same assembler; DA: different assembler. WF – Workflow number SA: same assembler; DA: different assembler. Samples are numbered in the order shown in Table 4. The supplemental data for the heatmaps are both the Binary and Identity excel files in the scripts repository at https://github.com/ErasmusMC-Bioinformatics/BenchAMRking-scripts/tree/main. (**b**) Heatmap representation of the identity of AMR genes detected in the workflows included in BenchAMRking. Colours represent different values of AMR gene identity between the different workflows. SA: same assembler; DA: different assembler. Samples are numbered in the order shown in Table 4. The supplemental data for the heatmaps are both the Binary and Identity excel files in the scripts repository at https://github.com/ErasmusMC-Bioinformatics/BenchAMRking-scripts/tree/main

The output of each WF was generated in a tabular format. For the visualization of the WF results, the output was concatenated and grouped using Python scripts (available in the BenchAMRking Github (Github repository of Erasmus/donny). R scripts to visualize the output data from the WFs - as shown in Figs. 2 and 3a and b - are also available in the BenchAMRking GitHub (https://github.com/ErasmusMC-Bioinformatics/BenchAMRking-script).

**Table 4:** Isolates and their online location for the ten whole genome bacterial sequences used in the pilot comparison of four BenchAMRking workflows and obtained from abritAMR [5]

*N*, Sample ID	Species	Reads Accession link and ID	Project Accession link and ID
1. SRR13803595	*E. coli*	https://www.ncbi.nlm.nih.gov/sra/SRS5393392	https://www.ncbi.nlm.nih.gov/bioproject/PRJNA565795
2. SRR13803509	*E. faecium*	https://www.ncbi.nlm.nih.gov/sra/SRS8336102	https://www.ncbi.nlm.nih.gov/bioproject/PRJNA565795
3. SRR6980278	*E. faecium*	https://www.ncbi.nlm.nih.gov/sra/SRS3155711	https://www.ncbi.nlm.nih.gov/bioproject/PRJEB11390
4. SRR14673537	*E. faecium*	https://www.ncbi.nlm.nih.gov/sra/SRR14673537	https://www.ncbi.nlm.nih.gov/bioproject/PRJNA856406
5. SRR14673481	*E. faecium*	https://www.ncbi.nlm.nih.gov/sra/SRS9084754	https://www.ncbi.nlm.nih.gov/bioproject/PRJNA565795
6. SRR14673267	*E. faecium*	https://www.ncbi.nlm.nih.gov/sra/SRS9084968	https://www.ncbi.nlm.nih.gov/bioproject/PRJNA565795
7. SRR10127028	*E. coli*	https://www.ncbi.nlm.nih.gov/sra/SRS5393392	https://www.ncbi.nlm.nih.gov/bioproject/PRJNA565795
8. SRR9734617	*K. pneumoniae*	https://www.ncbi.nlm.nih.gov/sra/SRR9734617	https://www.ncbi.nlm.nih.gov/bioproject/PRJNA529744
9. SRR15097991	*K. pneumoniae*	https://www.ncbi.nlm.nih.gov/sra/SRS9451555	https://www.ncbi.nlm.nih.gov/bioproject/PRJNA529744
10. SRR13803536	*S. aureus*	https://www.ncbi.nlm.nih.gov/sra/SRS8336075	https://www.ncbi.nlm.nih.gov/bioproject/PRJNA565795

### RO-Crate FAIR digital objects

RO-Crate, or Research Object Crate, is a format for storing research related files, datasets, and documents in a FAIR way [9]. For findability, RO-Crate contains metadata such as title, authors, date of creation, and other ID’s relevant for findability. All data, resources, and metadata are contained within the RO-Crate, ensuring accessibility. Interoperability and reusability were achieved by using the JSON-LD format, which is widely supported by most (bio-)informatics systems. The RO-Crates for each workflow are located in the corresponding Workflow Hub.

## Results

We have developed BenchAMRking (Fig. 1) to provide end-users and bioinformaticians with a suite of standardised AMR gene prediction workflows that have been replicated for use in the Galaxy environment. We have implemented four workflows: WF1 is an ISO certified AMR gene prediction workflow; WF2 – WF3 are examples of workflows developed by partners in the JPIMAR Seq4AMR and B2B2B networks, while WF4 is a well-characterised workflow for *Salmonella* spp. in human patients. All workflows were chosen to be representative of standardised AMR gene prediction analysis methodologies for multiple pathogens and for single pathogenic species. Furthermore, all the selected workflows were demonstrated to function properly using validation data sets. In the following sections, we outline the tools incorporated into the Galaxy toolshed and the steps in these individual workflows (WF1-4). The user can access and use all workflows and retrieve all FASTQ files (both primary data and contigs). The underlying code in our GitHub repository is accessible from the BenchAMRking landing page (https://erasmusmc-bioinformatics.github.io/benchAMRking/).

**Fig. 4:**
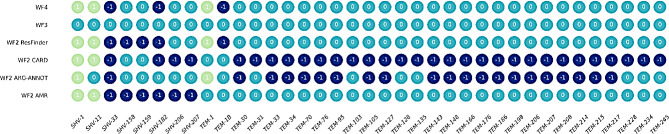
A comparison of the results obtained by WF1 (abritAMR) with those of WF2-4 via BenchAMRking. The AMR genes identified by both WF1 and WF2-4 are shown in light blue; those identified only by WF1 are shown in dark blue; those identified only by WF2-4 are shown in green

To illustrate the differences in AMR gene calling generated by the four different BenchAMRking workflows, a pilot study was performed using ten whole genome sequences obtained from abritAMR’s validation dataset [5], in two experiments (see Table 5). Accessions for the whole genome sequences are shown in Table 4. The output of the WFs is generated in a tabular format. For the visualization of the results, the output was concatenated and grouped using python scripts, with R scripts being used to visualize the output data (see https://github.com/ErasmusMC-Bioinformatics/BenchAMRking-scripts). Comparison of the results from the two experiments indicated limited concordance in the prediction of the AMR genes between the four WFs (Figs. 2 and 3a and b, and 4). Many of the discrepancies obtained were associated with different names (spelling variants) between AMR genes and different databases.

**Table 5:** Experiments performed using BenchAMRking platform

**EXPERIMENT 1**	
**Aim** - To check WF AMR predictions based on the use of the same assembler (SA)	
**Methodology** - WF2 Assemblies were used as an input of the four WF using ten samples (Table 4)	
**Tools** - Spades v3.15.5 / BenchAMRking	
**Samples** − 10 samples from abritAMR (Table 4)	
**EXPERIMENT 2**	
**Aim** - To check WF AMR predictions based on using a different assembler (DA)	
**Methodology** - WF2 and shovill assembler for 10 samples (Table 4)	
**Tools** - Spades v3.15.5 / shovill v1.0.4 / BenchAMRking	
**Samples** − 10 samples from abritAMR (Table 4)	

## Discussion and conclusions

BenchAMRking delivers easy and FAIR (Findable, Accessible, Interoperable, Reusable) access to both input data and standardised state-of-the-art AMR gene prediction workflows (WF1-4) in Galaxy. The workflows may be used in research and in diagnostic microbiology laboratories. Whilst BenchAMRking workflows are designed to be executed on Galaxy, the use of the Workflow Hub to generate a RO-Crate for each workflow ensures that they can also be executed in non-Galaxy based workflow applications. Thus, BenchAMRking goes beyond delivering reproducible workflow results that are comparable and available to the broader research community and the clinical field. The platform can contribute to the epidemiology and treatment of global AMR using WGS. As BenchAMRking is an open source and freely available platform, the authors hope that collaborations with interested colleagues will facilitate additional workflows and adaptations of the code and content beyond its current version. Our aim is to help democratize and promote a more comprehensive, standardised, and validated series of bioinformatics workflows for AMR gene prediction to help combat the current AMR pandemic. Finally, BenchAMRking is a tool whose feasibility is shown and described in this publication, examining over 500 AMR genes within 10 samples and 20 assemblies. More extensive studies using a broader and deeper range of international sequence data are currently being performed. Additional contributions and suggestions from international stakeholders interested in AMR, bioinformatics, workflow development and policy are welcome with the final goal of generating internationally agreed standards for gene sequence to AMR phenotype prediction workflows.

### Project link and requirements

- Project name: BenchAMRking.
- Project home page: https://erasmusmc-bioinformatics.github.io/benchAMRking/.
- Operating system: Windows and Linux.
- Programming Language: Python and R.
- License: GNU GPL.

## Electronic Supplementary Material

Below is the link to the electronic supplementary material.

Supplementary Material 1

Supplementary Material 2

Supplementary Material 3

## Data Availability

The datasets generated and/or analysed during the current study are available in the following repositories. 1) For the pilot comparison study of the four BenchAMRking workflows we used ten whole genome bacterial sequences obtained from abritAMR, and their accession details are listed in Table 4) All Galaxy wrappers developed are available for installation from the Galaxy Tool Shed (https://toolshed.g2.bx.psu.edu/). The workflows described in this publication are publicly available from the European Galaxy server, including published Galaxy histories (Table 3). All scripts used to generate the figures are available on GitHub (https://github.com/ErasmusMC-Bioinformatics/BenchAMRking-scripts).

## Abbreviations

AMR: Antimicrobial resistance
B2B2B AMRDx: Bench to Bedside to Business and Beyond: innovative solutions for AMR diagnostics
ISO: International Organisation for Standardisation
JPIAMR: Joint Programming Initiative on Antimicrobial Resistance
NGS: Next Generation Sequencing
Seq4AMR: JPIAMR Network for Integrating Microbial Sequencing and Applications for Antimicrobial Resistance
WF: Workflows
WGS: Whole genome sequencing
FAIR: Findable, Accessible, Interoperable, Reusable
RO-Crate: Research Object Crate
